# Beyond idiopathic short stature: opening a dialogue on nomenclature: **Response to the Letter to the Editor:** Growth failure of unknown etiology

**DOI:** 10.20945/2359-4292-2026-0114

**Published:** 2026-10-05

**Authors:** Alexander A. L. Jorge, Paulo F. Collett-Solberg, Margaret C. S. Boguszewski

**Affiliations:** 1 Unidade de Endocrinologia Genética (LIM25), Divisão de Endocrinologia, Hospital das Clínicas da Faculdade de Medicina da Universidade de São Paulo, São Paulo, SP, Brasil; 2 Disciplina de Endocrinologia, Departamento de Clínica Médica, Faculdade de Ciências Médicas da Universidade do Estado do Rio de Janeiro, Rio de Janeiro, RJ, Brasil; 3 Departamento de Pediatria, Universidade Federal do Paraná, Curitiba, PR, Brasil

Dear Editor,

We appreciate Professor Rapaport’s thoughtful Letter regarding our Brief Communication and his longstanding contribution to terminology for children with unexplained short stature or growth failure (^1^). His comments reveal substantial convergence with our proposal. Most importantly, we fully agree that every child with short stature or growth failure deserves appropriate clinical evaluation and, when necessary, longitudinal follow-up (^2^). Healthy Short Stature (HSS) was never intended to replace this principle or imply that short stature should not be investigated (^3^).

Professor Rapaport raises an important concern that “healthy” might be interpreted as meaning that no further attention to growth is required. This interpretation should be avoided. HSS is intended to describe the current clinical status after appropriate evaluation, not to provide a permanent certificate of health. The designation should follow adequate clinical, laboratory, and imaging evaluation, should not preclude genetic testing when indicated, and should be periodically reassessed (^3^). New clinical findings or an abnormal growth trajectory should prompt reconsideration.

His comments also highlight growth velocity as an important supporting feature. Growth velocity appropriate for age, sex, and pubertal stage-or sustained growth approximately parallel to the reference curve over 6-12 months-helps distinguish a short but normally growing child from one experiencing active growth failure (^4^,^5^). We deliberately avoided a specific height SDS cutoff because height is a continuous biological trait: increasing severity raises the probability of an underlying pathological or monogenic condition but does not, by itself, establish disease. In practice, very few children with severe short stature would be expected to fulfill all other proposed HSS criteria.

At the same time, we believe it is important to recognize explicitly that a child can be short and healthy. This point was central to our proposal. In clinical practice, children at the lower end of the height distribution may undergo extensive investigation, experience considerable family anxiety, and sometimes be regarded as having a disorder requiring treatment despite otherwise normal health and growth. The widespread use of ISS as a diagnostic label may unintentionally reinforce this medicalization of normal human height diversity. We therefore share Professor Rapaport’s appreciation of the older concept of “normal growth variants”, even though terminology and our understanding of growth biology have evolved substantially since that concept was introduced.

Recent efforts illustrate both progress and the need for further discussion. The International guideline on genetic testing of children with short stature proposed a more structured classification of short stature of unknown etiology, including “idiopathic isolated short stature” (^6^); however, reconsideration of nomenclature was not its primary purpose. Our Brief Communication was deliberately framed as a manifesto rather than a definitive consensus proposal. We do not assume that HSS will necessarily become the final terminology, and Professor Rapaport’s proposal of growth failure of unknown etiology also deserves consideration.

The challenge ahead is therefore to develop terminology that simultaneously achieves two objectives: not to medicalize or stigmatize healthy variation in human height, while never diminishing clinical vigilance for pathological growth. We hope that our proposal, Professor Rapaport’s thoughtful response, and further contributions from the pediatric endocrine community will stimulate the broader discussion needed to reach such a framework.

## Data Availability

datasets related to this article will be available upon request to the corresponding author.
